# The severity of postoperative complications after robotic versus laparoscopic surgery for rectal cancer: A systematic review, meta-analysis and meta-regression

**DOI:** 10.1371/journal.pone.0239909

**Published:** 2020-10-01

**Authors:** Yanlei Wang, Yanfei Liu, Gaoyang Han, Bo Yi, Shaihong Zhu

**Affiliations:** 1 Department of General Surgery, Third Xiangya Hospital, Central South University, Changsha, Hunan, China; 2 School of Nursing, Capital Medical University, You An Men, Beijing, China; 3 Department of Thoracic Surgery, Zhengzhou Central Hospital Affiliated to Zhengzhou University, Henan, China; Cleveland Clinic, UNITED STATES

## Abstract

**Objective:**

Robotic surgery (RS) has been increasingly used for the resection of rectal cancer, and its advantages over laparoscopic surgery (LS) have been demonstrated. However, few studies focused on the severity of postoperative complications. This study aimed to compared the postoperative complications within 30 days after RS over LS according to the Clavien-Dindo (C-D) classification.

**Methods:**

A literature research of PubMed, Embase, Cochrane Library and Web of Science were systematically performed. The studies comparing the complications of RS and LS for rectal cancer based on the C-D classification were enrolled. Primary outcomes were C-D grade III, IV, V, III-V (severe complications).

**Results:**

Seventeen studies (3193 patients) were included in the final analysis: 1554 underwent RS and 1639 underwent LS. The RS group was associated with significantly lower rates of severe complications (OR = 0.69, 95% CI 0.53–0.90, *P* = 0.005), C-D grade IV (OR = 0.69, 95% CI 0.53–0.90, *P* = 0.005), and anastomotic leak (OR = 0.66, 95% CI 0.48–0.91, *P* = 0.01). There was no significant difference in C-D grade III, C-D grade I, II, I-II (minor complications), overall complications, bleeding, wound complications, postoperative ileus, urinary retention, readmission, reoperation between two groups.

**Conclusions:**

Robotic surgery is safe for rectal cancer and may be an effective alternative to laparoscopic surgery, with lower rates of severe complications, C-D grade IV, and anastomotic leak. Further large randomized controlled trials are necessary to confirm this conclusion.

## Introduction

Since the laparoscopic surgery (LS) for colorectal cancer was first introduced in 1991 **[1]**, it has gained worldwide attention and now is considered as a standard operation. Randomized controlled trials (e.g., COREAN trial **[2]** and COLOR II trial **[3]**) demonstrated that the laparoscopic colorectal surgery involved a shorter hospital stay, less blood loss and postoperative pain compared with conventional open surgery. And these results do not compromise oncological outcomes **[2–4]**. However, the laparoscope has some innate limitations, such as two-dimensional view, limited range of motion, poor of dexterity, which requires a steep learning curve especially in the narrow pelvic cavity.

Robotic surgery (RS) provides several potential technical advantages, including three-dimensional vision, flexible endo-wristed instruments, improved ergonomics, and a stable camera platform **[5, 6]**. These advantages can translate into clinical benefits. Several meta-analyses for rectal cancer indicated that RS had favorable results over LS in terms of conversion, estimated blood loss, hospital stay and functional outcomes **[7–9]**. Furthermore, RS does not increase the rate of complications: The ROLARR study reported a comparable rate of complications between RS and LS (31.7% vs. 33.1%) **[10]**, consistent with other studies **[7, 8]**. Of note, a complication with different severity results in distinct symptoms. For example, a small anastomotic leak may accompany no symptoms, whereas a large anastomotic leak may lead to serious symptoms such as shock, sepsis and so on. Based on the severity of the complication, the patient is treated with various strategies such as conservative, surgical, endoscopic, or radiologic intervention and the patient suffer from a distinct experience. However, most of the reports just focused on the number of complications and did not take the severity of each complication into consideration. Only a few studies with small sample sizes involved this outcome. Large sample size research and systematic analysis are needed.

Therefore, to assess the safety of RS for rectal cancer, we performed a meta-analysis of published studies comparing robotic and laparoscopic surgery in term of postoperative complications, especially the severity of each complication according to the Clavien-Dindo (C-D) classification.

## Materials and methods

### Literature search strategy

We performed a systematic literature search of PubMed, Embase, Cochrane Library, and Web of Science on April, 2020. The search terms with a combination of medical subject headings (MeSH) and free-text words were as follows: (rectal neoplasms OR rectal cancer OR rectal tumor OR rectal adenocarcinoma OR rectum cancer OR rectum tumor OR rectum adenocarcinoma) AND (robotics OR robot OR robotic OR robotically OR robot-assisted OR robotic-assisted) AND (laparoscopy OR laparoscope OR laparoscopic). We manually screened the references from the articles selected to identify other potentially relevant researches. The PRISMA guidelines were followed for analysis of these studies **[11]**.

### Inclusion and exclusion criteria

The inclusion criteria were as follows: (i) patients with histologically diagnosed rectal cancer; (ii) comparative studies between RS and LS for rectal cancer, regardless of the study design (RCTs and non-RCTs); (iii) studies that clearly reported the grade of postoperative complications based on the C-D classification **[12]**; (iv) the most recent or the larger sample size studies were selected if studies reported on the same study population. Exclusion criteria were: (i) case reports, letters, comments, conference proceedings, review articles, meta-analyses, abstracts only; (ii) studies that reported postoperative complications without the C-D classification; (iii) studies including combined resection or Hartmann procedure; (iv) studies published in languages other than English.

### Data extraction and quality assessment

Two reviewers (WYL, LYF) independently searched the titles/abstracts and then the full-texts of the potential studies based on the inclusion criteria. The reviewers used a dedicated data form to extract variables from the included studies and cross-checked to reach a consensus. A third reviewer was involved to solve the disagreement. The quality of RCTs were assessed using Cochrane's tool with a total of 7 items **[13]**. The risk of bias was stratified into low (all items met), moderate (1–6 items met) and high (no items met) **[14]**. The quality of non-RCTs were assessed using the Newcastle Ottawa Scale (NOS) with a maximum score of 9 points (low quality:1–3 points, moderate quality: 4–6 points and high quality:7–9 points) **[15]**.

### Outcomes of interest

Only the postoperative complications within 30 days after surgery were considered. The complications were stratified into grade I-V according to the C-D classification, in which the grades I-II were considered as minor complications and the grades III-V were considered as severe complications. The primary outcomes include C-D grade III, IV, V, III-V (severe complications); the secondary outcomes include C-D grade I, II, I-II (minor complications), overall complications and individual complications (anastomotic leakage, bleeding, wound complications, abdominal abscess, ileus, urinary retention), reoperation and readmission.

### Statistical analysis

The statistical analyses were performed using the Review Manager software (Revman 5.3, The Cochrane Collaboration) and Stata/SE 12.0 (Stata Corp LP, Texas 77845). All variables were pooled using odds ratio (OR) with 95% confidence intervals (CI) and the analyses were performed using the Mantel-Haenszel method. Statistical heterogeneity was assessed using the *I*^2^ statistic. *I*^2^ <25, 25–50 and >50% was defined as low, moderate and high heterogeneity, respectively **[16]**. A fixed effects model was used when *I*^2^ <50%, otherwise the random effects model was used. Sensitivity analysis was performed to assess the robustness of the pooled results. Publication bias was quantitatively assessed by funnel plots for the primary outcomes. All *p* values were two-side and *p* < 0.05 was considered statistically significant.

Furthermore, a meta-regression analysis was performed to assess the potential effect of demographic and clinical variables (patient age, male gender, BMI) on the rate of severe complications.

## Results

### Identification of studies

**Fig 1:**
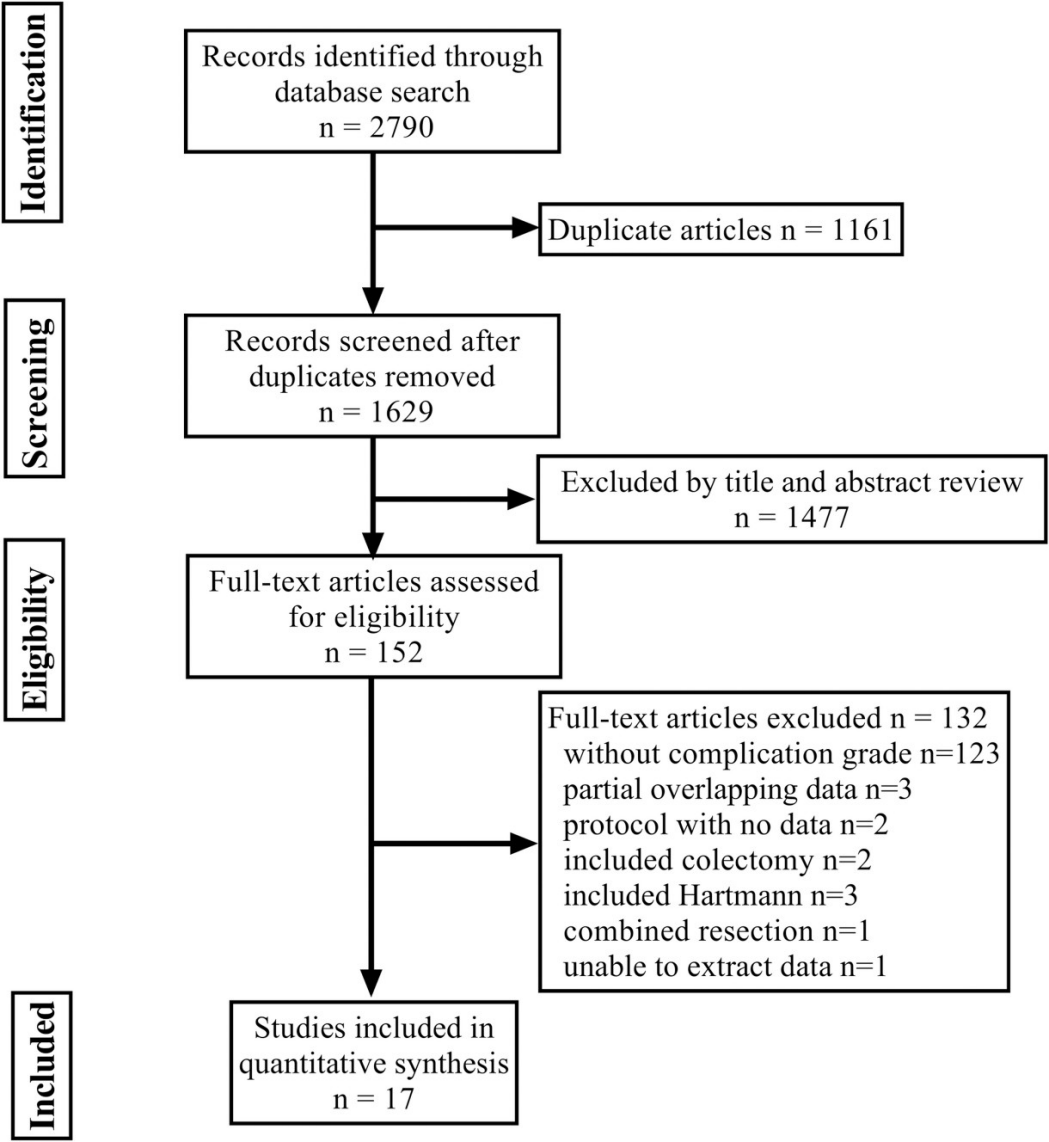
PRISMA diagram of the literature search.

The selection process of the study is demonstrated in **Fig 1**. A total of 2790 references were retrieved from the initial database search. After exclusion of duplicate and irrelevant references, 152 potential articles were retrieved. Seventeen articles were finally included in the meta-analysis after reviewing the full-texts, all of which were published within the last 9 years (2011–2020).

### Characteristics of included studies

**Fig 2:**
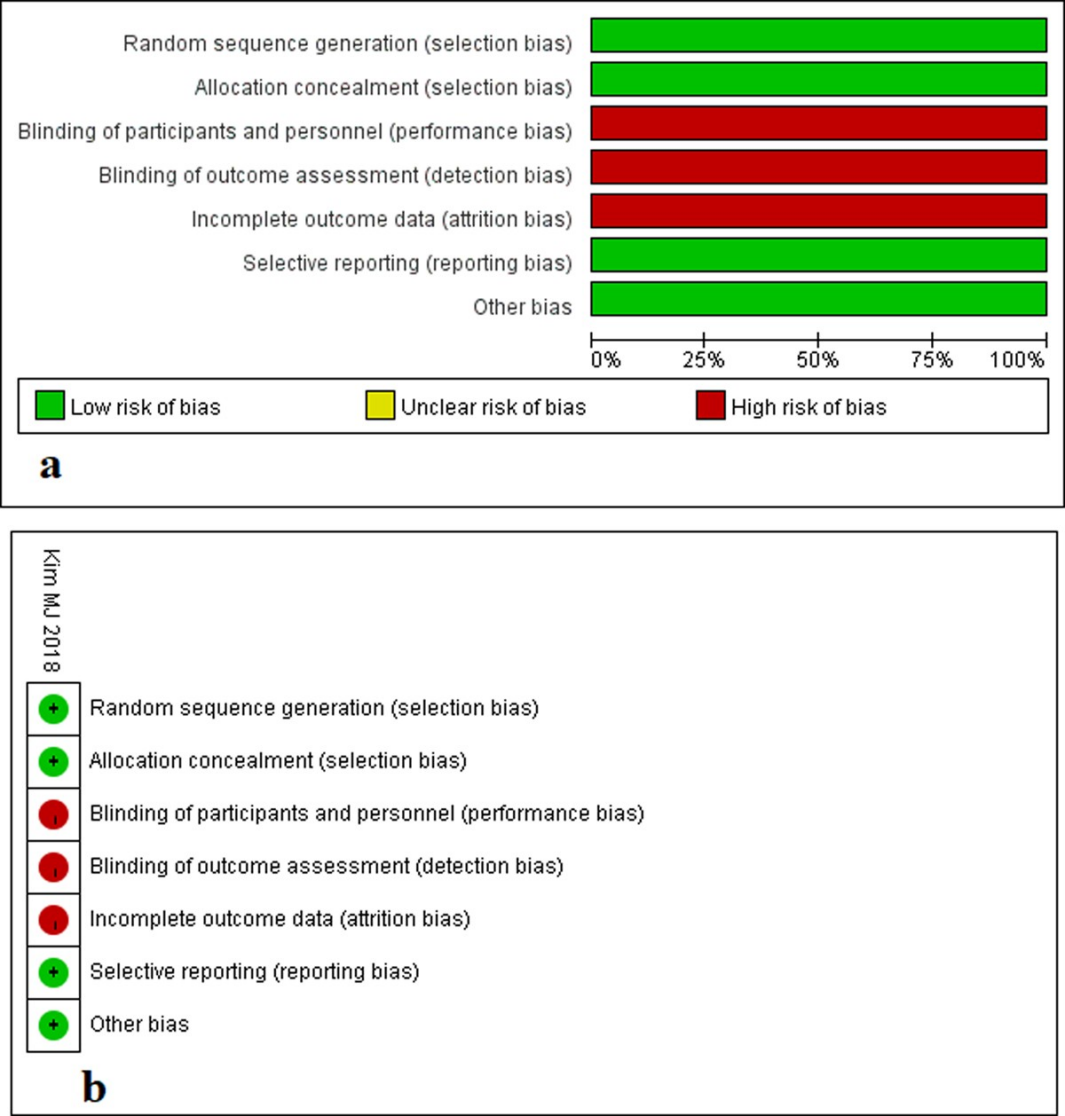
Quality assessment of RCT. **a** Risk of bias graph: judgments about each risk of bias item presented in RCT; **b** risk of bias summary: judgments about each risk of bias item for each RCT.

Among the 17 studies, one study was RCT **[17]**, 16 studies were non-RCTs **[18–33]** (13 retrospective studies and 3 prospective studies). These studies included 3193 patients, 1554 (48.7%) in the RS group and 1639 (51.3%) in the LS group. Male patients made up the majority of the studies in RS and LS groups, and the percentage ranged from 50.9 to 77.3%, 47.1 to 75.7%, respectively. The location of tumors were mostly within 10 cm from the anal verge (i.e., mid-low rectal cancer). The majority of patients were in TNM stage I, II, III, and minority in stage IV. The characteristics of included studies are summarized in **Table 1**. The only one RCT was considered with a moderate risk of bias (**Fig 2**). The NOS scores of the 16 non-RCTs ranged from 5 to 9 points, which were considered as moderate to high quality (**Table 1**).

**Table 1:** Characteristics of the included studies.

First author		Year	Country	Study design		Robotic technique		Study size (RS/LS)	Age (years) (RS/LS)			Male (%) (RS/LS)	CRT(%) (RS/LS)	
Allemann [18]		2016	Switzerland	RCNT		Hybrid		20/40	64 ± 12/65 ± 13			60.0/60.0	65.0/60.0	
Aselmann [19]		2018	German	RCNT		Total		44/41	61.1 ± 11.5/65.1 ± 12.0			59.1/58.5	20.5/19.5	
Baek SJ [20]		2013	Korea	RCNT		Total		47/37	58.0 ± 12.9/61.8 ± 12.8			66.0/75.7	42.6/5.4	
Feroci [21]		2016	Italy	RCNT		NR		53/58	66 (42–84)/66 (33–80)			50.9/72.4	49.1/43.1	
Galata [22]		2019	German	PCNT		Hybrid		18/33	60.0 ± 11.8/62.3 ± 13.7			55.6/63.6	61.1/60.6	
Ielpo [23]		2017	Spain	RCNT		NR		86/112	63.9 ± 9.5 /61.6 ± 11.9			55.8/59.8	75.6/77.7	
Kim JC [24]		2016	Korea	RCNT		Total		533/486	55 ± 9/58 ± 9			62.5/62.1	34.0/13.6	
Kim MJ [17]		2018	Korea	RCT		Total		66/73	60.4±9.7/59.7±11.7			77.3/71.2	77.3/79.5	
Mégevand [25]		2019	Italy	RCNT		Total		35/35	70 ^b^/66 ^b^			65.7/51.4	NR	
Park EJ [26]		2015	Korea	PCNT		Hybrid		133/84	59.2 ± 11.4/63.5 ± 11.2			64.7/71.4	11.3/11.9	
Park JS [27]		2011	Korea	RCNT		Hybrid		52/123	57.3±12.3/65.1±10.3			53.8/56.9	23.1/8.1	
Park JS [28]		2015	Korea	RCNT		Hybrid/total		106/106	59.6±10.8/61.7±9.6			70.8/67.0	64.2/56.6	
Quijano [29]		2020	Spain	PCNT		NR		81/104	64.0± 9.7/61.4± 10.7			54.3/47.1	74.1/77.9	
Ramji [30]		2016	Canada	RCNT		Hybrid		26/27	62.1±9.1/63.7±11.2			73.1/70.4	NR	
Shin [31]		2015	Korea	RCNT		Total		34/60	55±12.8/58±10.3			64.7/58.3	67.6/66.7	
Sugoor [32]		2019	India	RCNT		Total		84/84	48.3±15.5/49.2±14.8			72.6/69.0	67.9/65.5	
Tejedor [33]		2019	UK	RCNT		NR		136/136	68 (16) ^a^/69 (14) ^a^			55.9/55.9	20.6/16.2	
First author	BMI (kg/m^2^) (RS/LS)				Distance from AV (cm) (RS/LS)		TNM (I:II:III:IV) (RS/LS)			Newcastle-Ottawa Scale					
										Selection	Comparability			Outcome	
Allemann [18]	25.9 ± 9/24.2 ± 7				4.1 ± 1.7/4.8 ± 2.6		5:5:7:3/8:13:15:4			★★★★	★★			★★★	
Aselmann [19]	25.0 ± 3.8/25.7 ± 4.0				9.3 ± 3.2/8.9 ± 1.2		17:12:14:1/8:12:12:8			★★★★	★★			★★★	
Baek SJ [20]	23.4 ± 3.3/23.4 ± 2.7				4.4 ± 2.3/5.5 ± 3.7		22:8:10:1/13:9:8:3			☆☆★★	★★			★☆★	
Feroci [21]	24.6 (18–31)/24.6(19–37)				8 (4–12)/8 (3–12)		NR			★★★★	★★			★★★	
Galata [22]	26.0 ± 4.0/27.4 ± 5.5				8.5 ± 4.0/7.7 ± 3.3		5:5:7:1/12:9:9:3			★★★★	★★			★★★	
Ielpo [23]	26.1 ± 4.1/25.7 ± 3.4				6.9 ^b^/7.5 ^b^		NR			★★★★	★★			★☆★	
Kim JC [24]	24.1 ± 3.1/23.8 ± 3				5.6 ± 3.3/8.2 ± 3		191:129:170:0/204:104:150:0			★★★★	☆☆			★★☆	
Kim MJ [17]	24.1±3.3/23.6±3.0				NR		NR			☆☆★★	★★			★☆☆	
Mégevand [25]	24.6 ^b^ /25 ^b^				8 ^b^/8 ^b^		NR			★★★★	★★			★☆☆	
Park EJ [26]	23.1 ± 2.9/22.9 ± 2.8				NR		49:36:48:0/22:28:34:0			★★★★	★☆			★★★	
Park JS [27]	23.7±2.4/23.6±3.3				7.6±3.4/8.7±3.5		15:15:22:0/34:52:37:0			★★★★	★☆			★☆☆	
Park JS [28]	24.3±2.8/23.8±3.3				3.2±1.0/3.3±1.1		NR			☆☆★★	★★			★☆★	
Quijano [29]	26.0± 4.2/25.0± 5.4				7.1 ^c^/7.3 ^c^		NR			★★★★	★★			★☆★	
Ramji [30]	27.8±5.5/27.6±5.5				NR		NR			★★★★	★☆			★☆★	
Shin [31]	23.7±3.1/23.1±4.3				2.7±0.9/2.5±0.7		12:4:18:0/21:5:28:5			☆☆★★	★★			★☆★	
Sugoor [32]	22.8±4.0/23.1±3.0				NR		NR			★★★★	★★			★★★	
Tejedor [33]	27±5/27±6				6 (6) ^a^/6 (9) ^a^		43:41:51:0/43:41:51:0			★★★★	★★			★★★	

RS robotic surgery, LS laparoscopic surgery, BMI body mass index, AV anal verge, CRT chemoradiotherapy, RCT randomized controlled trial, PCNT prospective comparative non-randomized trial, RCNT retrospective comparative non-randomized trial, NR no record Continuous values are presented as mean ± standard deviation or median (range) if not indicated otherwise: ^a^ median/IQR, ^b^ median, ^c^ mean.

### Primary outcomes

#### Calvien-Dindo grade III

**Fig 3:**
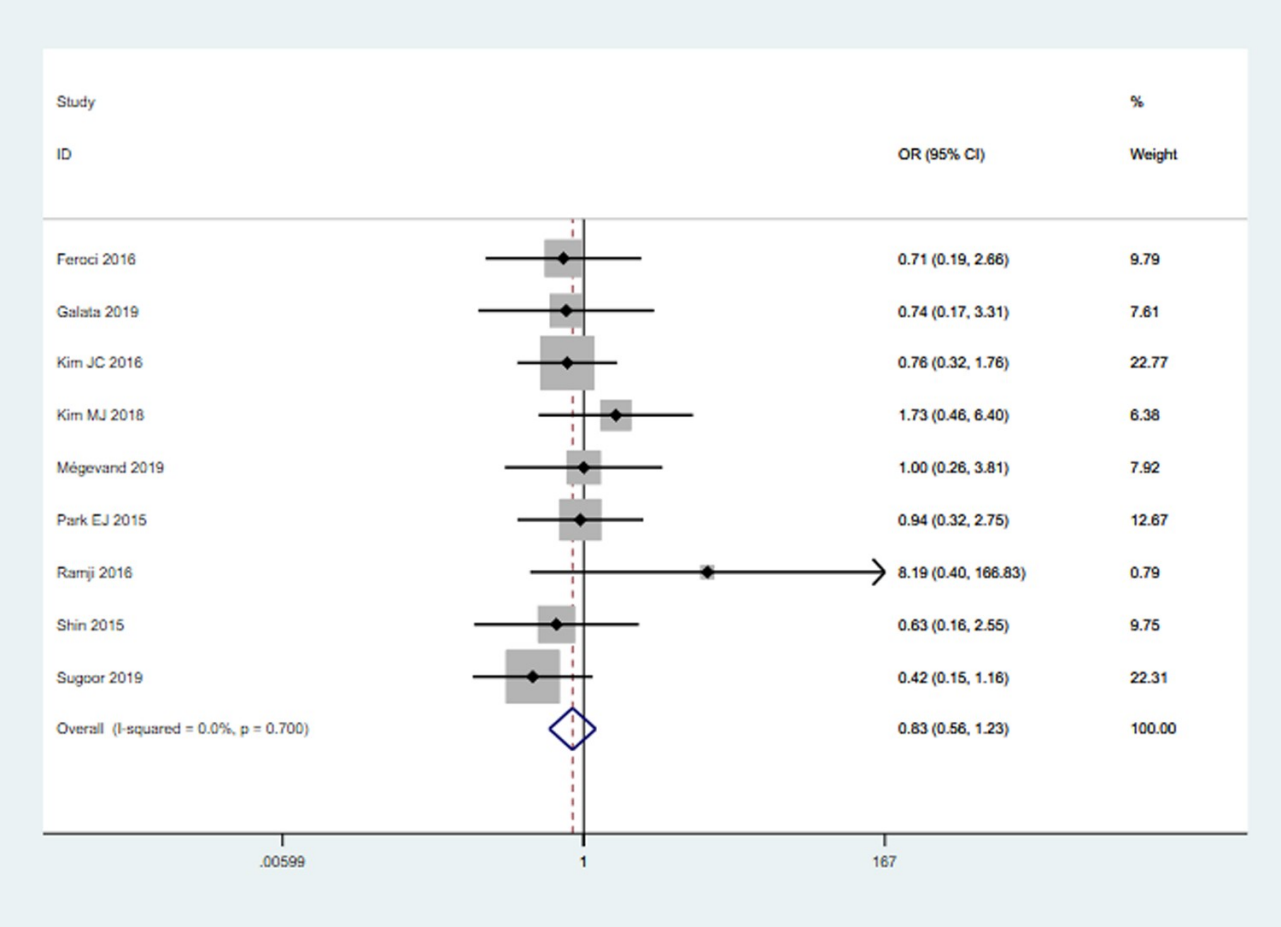
Pooled analysis for C-D grade III.

Nine studies (1922 patients) reported data on grade III complications. The rate of grade III complications was 5.0% (49/982) in RS group and 6.49% (61/940) in LS group. Pooled analysis showed that no significant difference was observed between the two groups (OR = 0.83, 95% CI 0.56–1.23, *P* = 0.35). There was no heterogeneity among the studies (*I*^2^ = 0%, *P* = 0.70) **(Fig 3)**.

#### Calvien-Dindo grade IV

**Fig 4:**
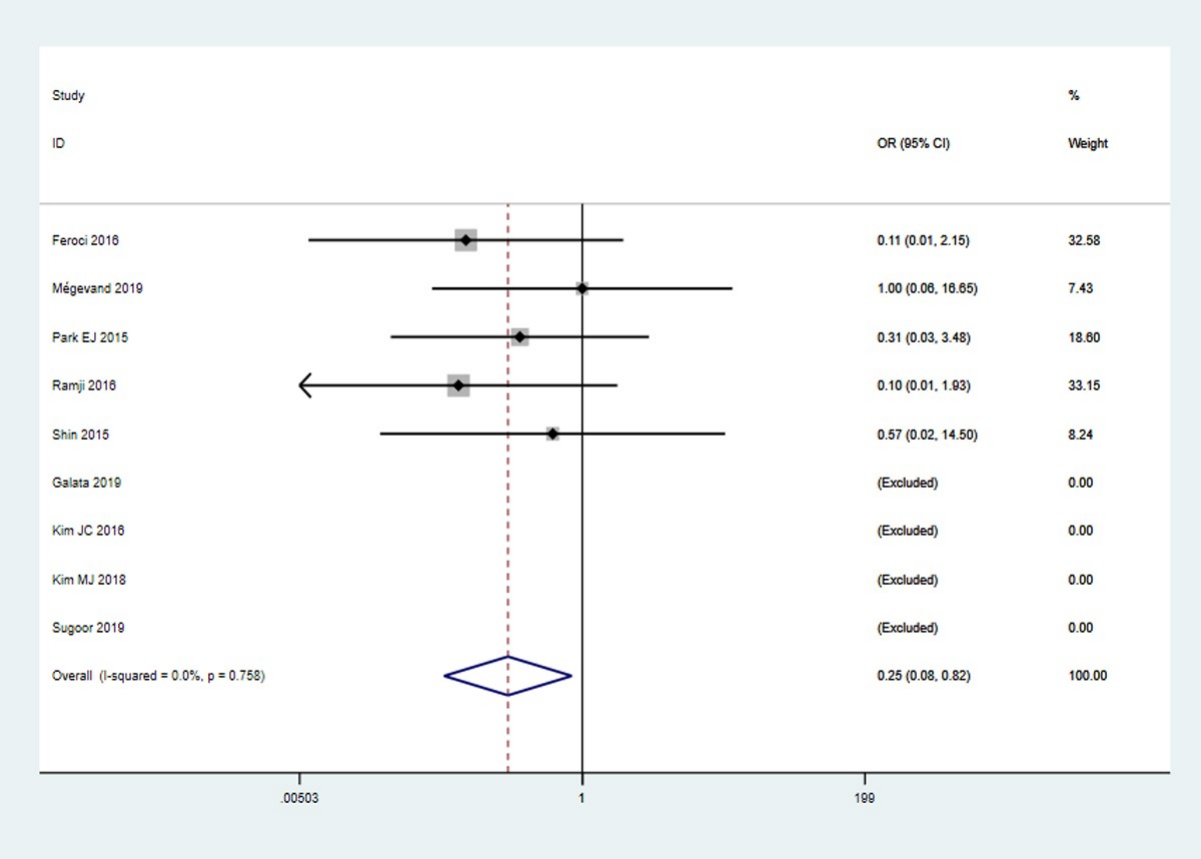
Pooled analysis for C-D grade IV.

There were 9 studies (1922 patients) reported the data on grade IV complications. The rate of this outcome was 0.20% (2/982) in RS group and 1.28% (12/940) in LS group. The RS group showed a lower rate of C-D grade IV (OR = 0.25, 95% CI 0.08–0.82, *P* = 0.02) as compared to LS group, with no significant heterogeneity (*I*^2^ = 0%, *P* = 0.76) **(Fig 4)**

#### Calvien-Dindo grade V

Among the 17 included studies, none of the patients experienced the grade V complication in both groups, and we did not solely take this outcome into meta-analysis.

#### Calvien-Dindo grade III-V (severe complications)

**Fig 5:**
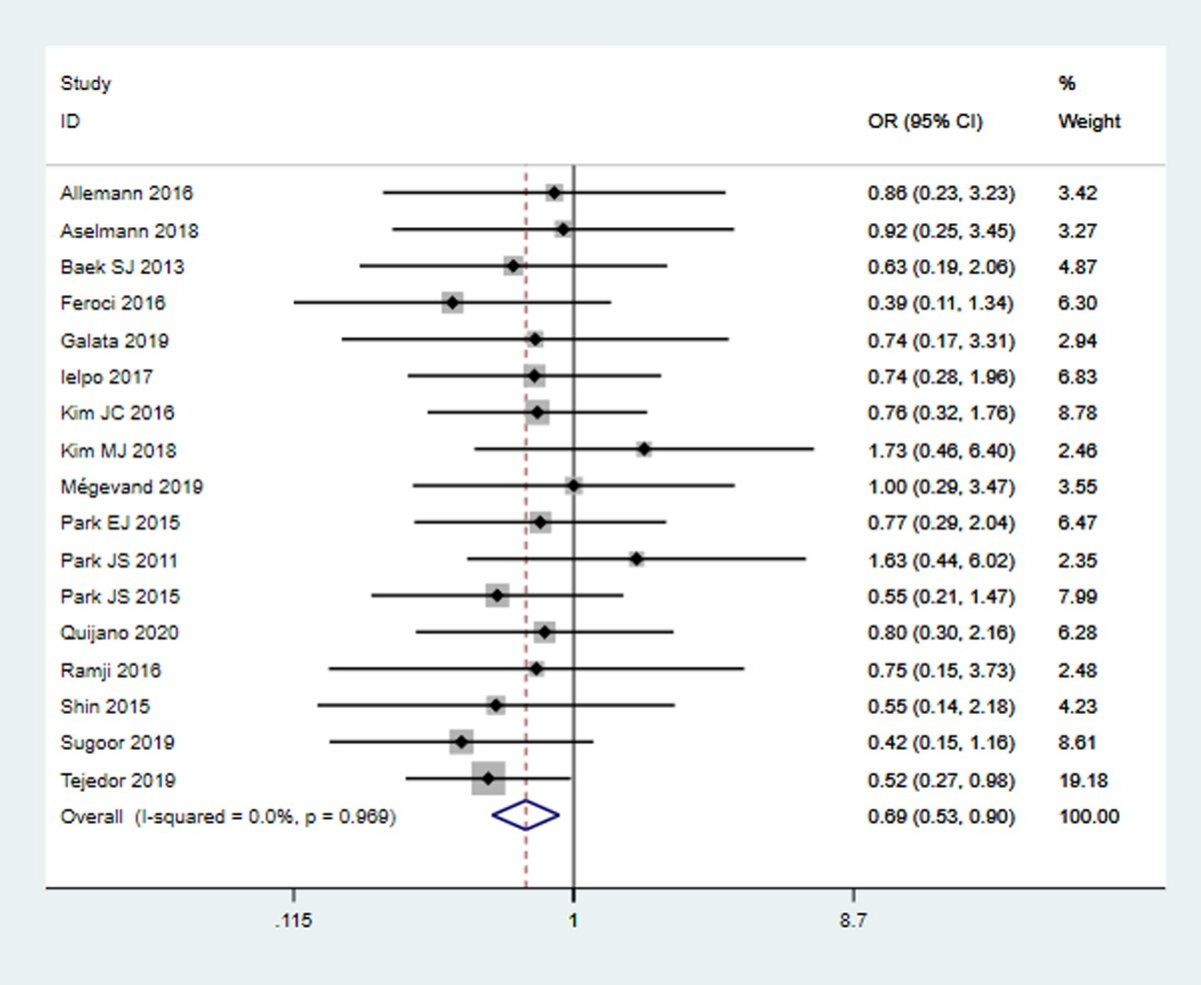
Pooled analysis for severe complications (grade III-V).

All 17 studies (3193 patients) reported this outcome. The rate of severe complication was 7.01% (109/1554) in RS group and 10.13% (166/1639) in LS group. Pooled analysis showed that RS group was associated with a significantly lower rate of severe complications as compared to LS group (OR = 0.69, 95% CI 0.53–0.90, *P* = 0.005), with no significant heterogeneity (*I*^2^ = 0%, *P* = 0.97) **(Fig 5)**.

### Secondary outcomes

#### Calvien-Dindo grade I

Eight studies (1754 patients) reported the grade I complications. The two groups were comparable in terms of this outcome (OR = 0.92, 95% CI 0.62–1.38, *P* = 0.69) and no heterogeneity was observed (*I*^2^ = 0%, *P* = 0.46) (**Table 2**).

**Table 2:** Summary of secondary outcomes for RS versus LS.

Outcome	Studies/Patients	*I*^2^ (%)	HG *p* value	OR (95% CI)	*p* value
C-D grade I	8/1754	0	0.46	0.92(0.62–1.38)	0.69
C-D grade II	8/1754	0	0.47	1.03(0.73–1.45)	0.86
C-D grade I-II	17/3193	22	0.19	1.04(0.96–1.12)	0.37
Overall complications	17/3193	16	0.27	0.89(0.74–1.08)	0.24
Anastomotic leak	14/2863	32	0.12	0.66(0.48–0.91)	**0.01**
Bleeding	8/1910	0	0.79	1.12(0.58–2.17)	0.74
Wound complications	10/2282	0	0.95	1.50(0.68–3.30)	0.32
Ileus	12/2317	0	0.89	0.81(0.54–1.20)	0.29
Urinary retention	8/2001	0	0.63	0.82(0.55–1.22)	0.32
Abscess	6/856	0	0.99	0.46(0.18–1.12)	0.09
Reoperation	6/473	0	0.78	0.53(0.27–1.04)	0.07
Readmission	8/786	30	0.20	0.80(0.48–1.34)	0.40

RS robotic surgery, LS laparoscopic surgery, C-D Clavien-Dindo.

#### Calvien-Dindo grade II

Eight studies reported the grade II complications, involving 898 patients in RS group and 856 patients in LS group. There was no significant difference in two groups (OR = 1.03, 95% CI 0.73–1.45, *P* = 0.86) and no heterogeneity was observed (*I*^2^ = 0%, *P* = 0.47).

#### Calvien-Dindo grade I-II (minor complications)

The grade I and II complications are considered as minor complications. All the 17 studies (3193 patients) reported this outcome and the rate was 29.21% (454/1554) in RS group and 31.73% (520/1639) in LS group. Notably, the rate of minor complications was high in four studies **[20, 27, 29, 33]**, with a range from 80.8 to 94.3%. The rate of minor complications was similar between two groups (OR = 1.04, 95% CI 0.96–1.12, *P* = 0.37), with low heterogeneity (*I*^2^ = 22%, *P* = 0.19).

#### Overall complications (grade I to V)

This outcome was reported by 17 authors, with a total of 3193 patients. The incidence of overall complications in RS group was 31.08% (483/1554) and 33.62% (551/1639) in LS group. The pooled data showed that the both groups was comparable (OR = 0.89, 95% CI 0.74–1.08, *P* = 0.24) and the heterogeneity was low (*I*^2^ = 16%, *P* = 0.27). It was worth noting that Quijano et al. **[29]** and Tejedor et al. **[33]** reported the rate of overall complications were 100%. The result was not affected with the two studies excluded.

#### Anastomotic leak

Fourteen studies reported the data on anastomotic leak. The incidence of anastomotic leak was 4.76% (67/1409) in RS group and 7.36% (107/1454) in LS group. The RS group was with a lower rate of anastomotic leak than LS group (OR = 0.66, 95% CI 0.48–0.91, *P* = 0.01), with no significant heterogeneity (*I*^2^ = 32%, *P* = 0.12).

#### Bleeding

Eight studies reported bleeding as an outcome. The site of bleeding included anastomotic stoma or pelvic cavity. The pooled data showed no significant difference was observed between robotic group (1.89%, 18/950) and laparoscopic group (1.77%, 17/960) (OR = 1.12, 95% CI 0.58–2.17, *P* = 0.74), with no significant heterogeneity among the studies (*I*^2^ = 0%, *P* = 0.79). Of the 8 studies, 3 studies **[19, 25, 26]** clearly reported the site of bleeding in the anastomotic stoma, with an incidence of 3.30% (7/212) in RS group and 5.63% (9/160) in LS group. The meta-analysis showed no significant difference (OR = 0.64, 95% CI 0.23–1.77, *P* = 0.39) and no heterogeneity (*I*^2^ = 0%, *P* = 0.81).

#### Wound complications

Ten studies reported the data with wound complication, in a total of 2282 patients. The incidence of wound complication in RS and LS groups were 1.00% (13/1304), 0.82% (8/978), respectively. There was no significant difference in both groups (OR = 1.50, 95% CI 0.68–3.30, *P* = 0.32) and no heterogeneity was observed (*I*^2^ = 0%, *P* = 0.95).

#### Postoperative ileus

Twelve studies reported the data on postoperative ileus, in 2317 patients. The incidence of postoperative ileus was 3.94% (45/1141) in RS group and 4.93% (58/1176) in LS group. The pooled analysis demonstrated no significant difference in two groups (OR = 0.81, 95% CI 0.54–1.20, *P* = 0.29) and there was no between-study heterogeneity (*I*^2^ = 0%, *P* = 0.89).

#### Urinary retention

The complication of urinary retention was reported by 8 authors with 2001 patients included. The incidence of urinary retention was 4.76% (47/988) in RS group and 5.53% (56/1013) in LS group. The pooled analysis demonstrated no significant difference in two groups (OR = 0.82, 95% CI 0.55–1.22, *P* = 0.32). No between-study heterogeneity was observed (*I*^2^ = 0%, *P* = 0.63).

#### Abdominal abscess

Abdominal abscess was evaluated in 6 studies (856 patients), with an incidence of 1.42% (6/424) in RS group and 3.94% (17/432) in LS group. The meta-analysis demonstrated comparable results in both groups (OR = 0.46, 95% CI 0.18–1.12, *P* = 0.09).

#### Reoperation

The rate of reoperation was recorded in 6 studies (473 patients). There is no significant difference between groups (OR = 0.53, 95% CI 0.27–1.04, *P* = 0.07) with no between-study heterogeneity (*I*^2^ = 0%, *P* = 0.78).

#### Readmission

Eight studies (786 patients) provided information on readmission, with a rate of 7.56% (27/357) in RS group and 9.32% (40/429) in LS group. Under a fixed effects model, the rate of readmission was similar between groups (OR = 0.80, 95% CI 0.48–1.34, *P* = 0.40) with moderate heterogeneity (*I*^2^ = 30%, *P* = 0.20).

### Sensitivity analysis and publication bias

**Fig 6:**
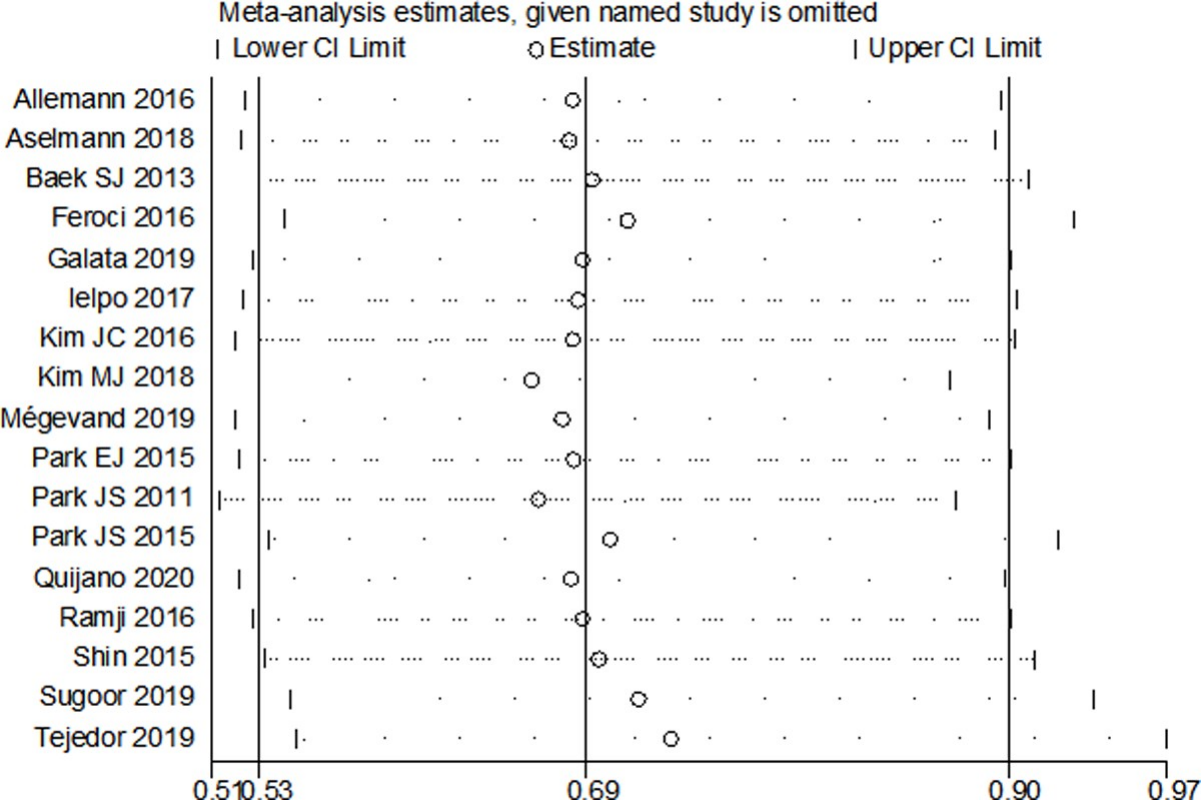
Sensitivity analysis of severe complications after RS vs. LS.

Sensitivity analysis was performed to investigate the potential sources of heterogeneity and the robustness of the outcomes. After excluding studies one by one, we found that the individual study did not influence the outcomes (**Fig 6**), except for the C-D grade IV, anastomotic leak and reoperation. In terms of the C-D grade IV, after removal of the study of Feroci et al. **[21]** or Ramji et al. **[30]**, the result materially altered, showing no statistically difference between groups. The similar result was also be observed in the outcome of anastomotic leak with an insignificant declining of heterogeneity, when omitting the study of Kim JC et al. **[24]** or Tejedor at al. **[33]**. In regard to reoperation, when the study of Allemann et al. **[18]** was excluded, the result was affected (OR = 0.44, 95% CI 0.21–0.94, *P* = 0.03), favoring the RS group.

**Fig 7:**
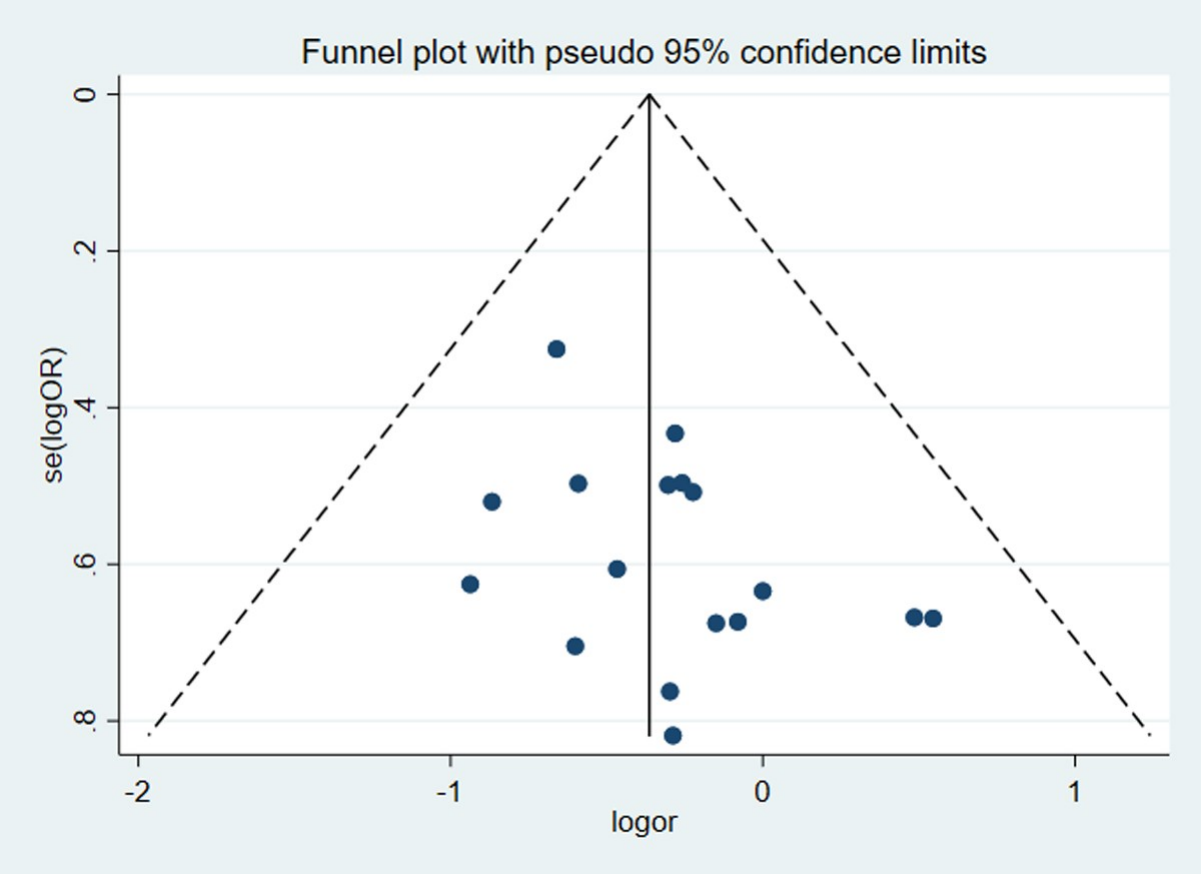
Funnel plot of the studies reporting on the rate of severe complications.

A funnel plot was used for assessing the publication bias for all of the outcomes. None of the studies lay outside the limits of the 95% CIs and all of the studies equally distributed on the vertical axis, indicating no obvious publication bias **(Fig 7)**.

### Meta-regression analysis

Meta-regression analysis showed that patient age (*P* = 0.779), gender (*P* = 0.986), BMI (*P* = 0.559) did not significantly impact the rate of severe complications.

## Discussion

In spite of the technical advantages mentioned above, robotic surgery still has some drawbacks including the limited range of movement of the robotic arm, longer operation time and higher cost of the system **[4]**. The most obvious disadvantage is the lack of tactile feedback, so an excessive force may be exerted on the tissue and subsequently a complication may occur **[34]**, although the surgeon’s experience can compensate for this situation. A complication is a crucial indicator for assessing the quality of operation, which is not only associated with increased length of hospital stay and medical expenditure, but also increases painful patient experience and sometimes even life-threatening **[35]**. A large number of studies only payed attention to the number of complications, whereas the severity of complications was ignored. The well-standardized system known as the C-D classification has been confirmed as an effective measure to evaluate the severity of postoperative complications **[12]**, which is stratified into five grades ranging from grade I (mild) to grade V (severe) according to the degree of intervention. To the best of our knowledge, this is the first systematic and comprehensive review pertaining to postoperative complications of RS versus LS using the C-D classification. In this meta-analysis, the results indicated some benefits for RS versus LS and suggested that the safety of robotic surgery was comparable to laparoscopic surgery, with a lower incidence of severe complications, C-D grade IV and anastomotic leak.

A severe complication (C-D grade ≥ III) is treated with surgical, endoscopic, or radiologic intervention. In our study, the incidence of severe complications in robotic surgery tended to be lower than laparoscopic surgery (7.01% vs. 10.13%), with an absolute risk reduction of 31%. This benefit attributed to the technical advantages of surgical robot. The 3D vision imaging system contributed to a more accurate spatial orientation; motion scaling allowed for a more precise manipulation, and the articulated instruments with seven degrees of freedom increased the dexterity of the instruments. All such characteristics enhanced the process of TME in a narrow pelvic cavity, thus reducing the occurrence of a severe complication. Based on five non-RCTs, Lee et al. **[36]** performed a similar meta-analysis on severe complications after robotic surgery over laparoscopic surgery for rectal cancer. The incidence was 9.52% (26/273) in robotic surgery and 11.39% (27/237) in laparoscopic surgery. Lee et al. failed to show a significant difference in the two groups and the fact that Lee et al. especially looked at intersphincteric resection for low rectal cancer may contribute to the different result from us.

The overall postoperative complications have been evaluated in recent literatures. Similar to our result, some articles reported a comparable outcome in two techniques **[7, 8, 10, 37, 38]**. However, other articles **[39–41]** showed a significant lower complication rate in robotic surgery. Sun et al. **[39]** specially compared the low anterior resection for rectal cancer. After pooling the data of one RCT and 6 non-RCTs, the rate of overall complications was 16.5% (52/324) in robotic surgery and 21.7% (60/276) in laparoscopic surgery (OR = 0.65, 95% CI 0.43–0.99, *P* = 0.04), with no heterogeneity (*I*^2^ = 0%, *P* = 0.52). Similarly, Cui et al. **[40]** analyzed the data of nine non-RCTs, showing a rate of 13.5% (64/473) in robotic surgery and 22.7% (108/476) in laparoscopic surgery. The robotic surgery was with a lower rate of overall complications (OR = 0.58, 95% CI 0.41–0.83, *P* = 0.003) with no heterogeneity (*I*^2^ = 0%, *P* = 0.95). The discrepant results might originate from the following reasons: (i) The preoperative baseline characteristics such as age, male, BMI, tumor location and tumor stage were not equal in each study. For example, in our study, male patients accounted for the majority of the study population; (ii) Different types of operation were analyzed, which included (high/low) anterior resection, (total/partial) mesorectal resection, intersphincteric resection, abdominoperineal resection. Even emergency or combined resections were included in previous studies. (iii) Different robotic approaches (hybrid or total) were applied; (iv) Different specimen retrieved techniques (trans-anal or mini-laparotomy).

Anastomotic leak is the most common and critical complication after rectectomy. Current study reported that robotic surgery was associated with a lower rate of anastomotic leak than laparoscopic surgery (4.76% vs. 7.36%), which was inconsistent with previous literature **[8, 10, 36, 38]**. We found that robotic surgery was with a lower rate of anastomotic leak in only three of the fourteen involved studies **[24, 32, 33]**, and the both groups showed a significant difference in patient characteristics such as age, tumor distance from anal verge, surgical procedure, and ileostomy. Some of these factors were the independent risk factors of anastomotic leakage **[42, 43]**, which may contribute to this result to a certain extent. Furthermore, sensitivity analysis showed that the pooled result was not robust and influenced by the individual study. Therefore, the conclusion regarding anastomotic leakage should be made cautiously. Comparable results were found in terms of bleeding, postoperative ileus, urinary retention, abdominal abscess, reoperation and readmission, which indicated equivalent safety and efficacy of robotic surgery, powerfully promoting its widespread in the world.

Notably, we excluded the studies involving Hartmann procedure (HP) **[35, 44, 45]**. For colorectal cancer, HP was primarily performed for patients with serious comorbidities and/or a challenging local situation in abdominal cavity (severe inflammation/ peritonitis/sepsis due to obstructed/perforated tumors) **[46, 47]**. All these situations were related to a high rate of complication, as mentioned by Jonker et al. **[48]**. He included 1728 patients of rectal cancer and reported a high morbidity of 40% after HP. Therefore, the three studies were excluded to control the confounding factors. Also, we attempted to incorporated the three studies in meta-analysis and found that the robotic surgery was still with a lower rate of severe complications.

This meta-analyses had some limitations. First, although all of the included studies were with moderate to high quality, the inherent property of the non-randomized studies such as the unequal characteristics of patients and the different experiences of surgeons, biased the interpretation of the results to some extent. Second, even though no heterogeneity in most of the outcomes, none of the studies made any adjustment for possible confounding factors, which may result in a high risk of selection bias. Third, the pooled studies included several types of procedures for rectal cancer and there was no access to the raw data, so no subgroup analysis was made. Trials distinguishing the different types of procedures are necessary to control the bias. In addition, we only compared the short-term complications and the long-term complications should also be involved in future studies.

## Conclusion

We found that robotic surgery was with similar short-term complications than laparoscopic surgery except severe complications, C-D grade IV and anastomotic leak. Therefore, the robotic approach can be safely applied in rectal cancer and may be an alternative treatment to overcome difficulties in the narrow pelvic cavity. Prospective randomized trials such as the ROLARR trial are needed to further compare the complications, including short-term and long-term complications according to the C-D classification.

## Supporting information

S1 Appendix PRISMA checklist. (DOC)

S2 Appendix PubMed search strategy. (DOCX)
