# Supplementary material for: The severity of postoperative complications after robotic versus laparoscopic surgery for rectal cancer: A systematic review, meta-analysis and meta-regression
Source: PLoS One. 2020 Oct 1;15(10):e0239909. doi: 10.1371/journal.pone.0239909 (PMC7529204; doi:10.1371/journal.pone.0239909)
Supplement: S2 Appendix — (DOCX) [file pone.0239909.s002.docx]

**PubMed search strategy**

| **#** | **search term** | **Result** |
| --- | --- | --- |
| #1 | (((((rectal cancer[Title/Abstract]) OR rectal adenocarcinoma[Title/Abstract]) OR rectum cancer[Title/Abstract]) OR rectum tumor[Title/Abstract]) OR rectum adenocarcinoma[Title/Abstract]) OR rectal tumor[Title/Abstract] | 24302 |
| #2 | "Rectal Neoplasms"[Mesh] | 46763 |
| #3 | #1 OR #2 | 53937 |
| #4 | "Robotics"[Mesh] | 27170 |
| #5 | ((((robot[Title/Abstract]) OR robotic[Title/Abstract]) OR robotically[Title/Abstract]) OR robot-assisted[Title/Abstract]) OR robotic-assisted[Title/Abstract] | 37846 |
| #6 | #4 OR #5 | 43782 |
| #7 | "Laparoscopy"[Mesh] | 96624 |
| #8 | (laparoscopic[Title/Abstract]) OR laparoscope[Title/Abstract] | 106198 |
| #9 | #7 OR #8 | 129843 |
| #10 | #3 AND #6 AND #9 | 529 |
